# Bio-Based Phosphate-Containing Polyester for Improvement of Fire Reaction in Wooden Particleboard

**DOI:** 10.3390/polym15051093

**Published:** 2023-02-22

**Authors:** Ingemar Svensson, Amaia Butron, Maddalen Puyadena, Alba González, Lourdes Irusta, Aitor Barrio

**Affiliations:** 1 TECNALIA, Basque Research and Technology Alliance (BRTA), 20730 Azpeitia, Spain; 2 POLYMAT, Department of Polymers and Advanced Materials, Physics, Chemistry and Technology, University of the Basque Country (UPV/EHU), 20080 Donostia-San Sebastián, Spain

**Keywords:** polyester, phosphate, particleboard, fire reaction, cone calorimeter

## Abstract

A new phosphate-containing bio-polyester based on glycerol and citric acid was synthesized and evaluated as fire-retardant (FR) in wooden particleboards. Phosphorus pentoxide was used to first introduce phosphate esters in the glycerol followed by esterification with citric acid to produce the bio-polyester. The phosphorylated products were characterized by ATR-FTIR, ^1^H-NMR and TGA-FTIR. After polyester curing, they were grinded and incorporated in laboratory produced particleboards. The fire reaction performance of the boards was evaluated by cone calorimeter. An increased char residue was produced depending on the phosphorus content and the THR (Total Heat Release), PHRR (Peak of Heat Release Rate) and MAHRE (Maximum Average of the Rate of Heat Emission) were considerably reduced in presence of the FRs. Highlights: Phosphate containing bio-polyester as fire retardant in wooden particle board; Fire performance is improved; Bio-polyester acts in the condensed and gas phases; Additive effectiveness similar to ammonium polyphosphate.

## 1. Introduction

With growing environmental concerns and limited availability of fossil resources, interest in biomass resources is at an all-time high [1,2,3]. Wood, as a sustainable resource, has been extensively employed as a building and decoration material because of its excellence strength-to-weight ratio [3,4]. As a result of excessive deforestation of natural forests and increasing demand for wood, the use of particleboards could help to mitigate the gap between supply and demand [3]. Particleboard, also known as chipboard, is obtained by mixing wood particles or chips with an adhesive (usually based on formaldehyde: urea-formaldehyde (UF), phenol-formaldehyde (PF) or melamine-formaldehyde (MF) [5,6]) and applying pressure and heat [5,6,7]. Although the main components are wood particles and adhesive, they can also contain fiber (particle) and additives or fillers [5,6]. These wood particles arise from forest thinning and timber waste from peeler cores, shavings and mill waste [6,8]. Even if wood residues are employed, significant cost reduction could be obtained using environmentally friendly agricultural biomass, recycled wood waste and by-products for particleboards formation, as state by S. H. Lee et al. [6]. In their work, it was demonstrated that alternative raw materials-based particleboards exhibit similar or even better mechanical properties than conventional wood-based particleboards.

Similarly to wood from natural forests, particleboards exhibit some undesirable properties, such as flammability, which strongly limit their use in construction [3]. F. Richter et al. showed with their work that engineered wood has three burning modes depending on the oxygen concentration (pyrolysis < 4 vol% O_2_, smoldering 4–15 vol% O_2_ and flaming > 15 vol% O_2_), among which smoldering presents a problem for timber construction [4]. To protect engineered wood products against fire, large amounts (10–30%) of halogenated salts, phosphorus or boron are commonly incorporated. To give one example, L. Zhang et al. were able to improve the thermal behavior and flame retardancy of poplar wood up to 217% by impregnating with boric acid and ammonium dihydrogen phosphate [3]. Although in this last work the wood was impregnated, these compounds can be incorporated in several ways. For example, K. Yue et al. showed that the incorporation of gypsum particles to wood to form a composite resulted in fire resistance properties as good as applying gypsum as sheathing [9]. However, S. Medved et al. described how important it was to ensure that the fire retardant was on the front line against fire, whether it was incorporated in the production of the boards or was sprayed at the finished panels [10].

Anyhow, for long-term use of the boards in outdoor conditions, there is the problem of leaching of the incorporated fire-retardant salts into rainwater and the corresponding toxicity issues of the lixiviate. In addition, the mechanical strength and surface properties of fire retardant wooden boards can be inferior to those of non-fire retardant boards [11]. Additionally, there are environmental and toxicity concerns about halogen and boron containing fire retardants. Recent restrictions are imposed by REACH and RoHS regulations for the use of halogenated compounds [12,13]. The use of ammonium salts and boron compounds are being restricted (under review in the EU), ammonium salts could release ammonia gas that is toxic for indoor use and boron is a reproductive inhibitor.

It is, therefore, of research interest to find alternative fire-retardant substances, especially in the grafting of phosphorus-containing molecules in (bio)polymers to reduce the risk of leaching and to improve the efficiency, so as to lower the percentage used in the final product. The review by Illy et al. [14] gives an important overview of phosphorylation of bio-based compounds such as polysaccharides, bio-phenols, triglyceride oils and hydroxyl acid compounds. Some examples deal with the obtaining of reactive monomers containing phosphorus for acrylic, phenolic, and epoxy resin applications. Some examples are summarized in Table 1. With polylactic acid (PLA) biopolyester, phytic acid could be applied using a pad-dry-curing technique that exhibited good fire resistant performance according to the limiting oxygen index (LOI) and vertical combustion test results, in addition to higher carbon residues and low heat release capacity [15]. Another approach would be to blend PLA with phosphorylated lignin in Brabender mixer as an additive flame-retardant [16]. Apart from PLA, bio-based polyesters with phosphorus could be obtained following different strategies. One such route involves the reaction of cellulose-origin 2,5-furandicarboxylic acid (FDCA) with 1,6-hexanediol (HDO) and 2-carboxyethyl (phenyl)phosphinic acid (CEPPA) to obtain poly(hexamethylene 2,5-furandicarboxylate) (PHFC), leading to a substantial increase in the LOI value [17]. Another option would be to obtain poly(butylene terephthalate) (PBT) employing phosphorized linseed oil and downstream corn oil obtained from ring opening reaction of the epoxidized vegetable oils, which seems to need small amounts of modified oils (10 wt%) to be incorporated in order to obtain UL-94 V-O rate [18]. Similarly, by incorporation of phosphorus in polyols from epoxidized soybean oil and reacting with polymeric isocyanate, rigid polyurethane foam with improved char and reduced peak heat release rate and total heat was produced [19]. The option of incorporating into polyurethane foams a reactive flame-retardant capable of blending with limonene dimercaptan has also been studied, resulting in polyurethane foams with high flame retardancy and superior physico-mechanical properties [20]. Phosphorus was possible to incorporate to epoxy resins by lignin derivative vanillin, coupling it with diamines and diethyl phosphite followed by reaction with epichlorohydrin, which results in outstanding intumescent and dense char-forming ability [21]. Not only vanillin but also guaiacol proved to be suitable for this purpose by making them react with 9,10-dihydro-9-oxa-10-phosphaphenanthrene-10-oxide (DOPO), which could be epoxidized with epichlorohydrin for further blending with diglycidyl ether of bisphenol A (DGEBA) and cured with 4,4-diaminodiphenylmethane (DDM). The obtained epoxy resin showed excellent fire resistance properties, to such an extent that it reached a UL-94 V-0 value when only 1.27 wt% phosphorus was incorporated [22]. Phosphorus-containing epoxy resin can also be obtained by modifying the epoxy monomer with phloroglucinol from fruit threes to obtain triglycidyl phloroglucinol and curing with decane-1,10-diamine or difurfurylamine. By means of this strategy, a decrease in thermal stability is obtained, but an increase in carbonization yield is observed at elevated temperatures [23]. In addition, poly(styrene) (PS) could be copolymerized with exo-5-(diphenylophosphato)isosorbide-2-endo-acrylate to provide 1–2 wt% of phosphorus, leading to a reduction of 50% in peak heat release rate for combustion. This last compound was obtained by making isosorbide react with diphenylchlorophosphate, and further incorporation of the vinyl group with acryloyl chloride [24].

In light of the possibility that incorporation of phosphorus in the polymer chain could improve the fire proofing properties, in the present work, a novel non-toxic bio-polyester was synthesized using phosphorus pentoxide (P_2_O_5_) as a phosphorylation reagent. The aim of this study was to prepare a phosphate containing bio-based polyester, based on glycerol and citric acid, and to use this as a fire retardant in wooden particle boards. The efficiency as FR was demonstrated by cone calorimeter test and compared with commercial additives like ammonium polyphosphate (APP).

## 2. Materials and Methods

### 2.1. Materials

Phosphorus pentoxide (P_2_O_5_), citric acid and glycerol (reagent grade) were supplied by Scharlau. A 65% dry content urea-formaldehyde resin (Q-125R) from Kronospan (Spain) was used. The commercial fire retardant used as reference was Clariant’s APP (Exolit AP422). All reagents were used as received.

### 2.2. Preparation of Phosphate Containing Polyester

**Scheme 1:**
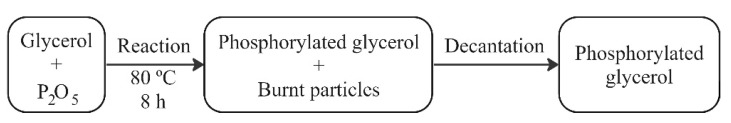
Flowchart of the preparation of phosphate containing glycerol.

**Figure 1:**
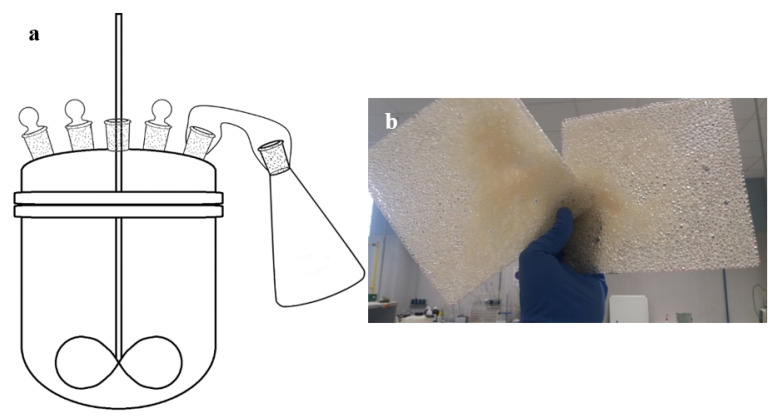
(**a**) Scheme of the reactor setup for glycerol phosphorylation. (**b**) Oven-cured phosphate-containing polyester.

First, the phosphorylation of glycerol was performed similarly to Bocz et al. [25] (see Scheme 1 and Scheme S1). First, 202.6 g (2.2 mol) of glycerol in reaction PG1 (3:1 glycerol to P-atom ratio) and 101.3 g (1.1 mol) in reaction PG2 (1.5:1 glycerol to P-atom ratio) were cooled (0 °C) in a stirred glass reactor. Then, 52.0 g of P_2_O_5_ (0.36 mol) was slowly added to the reactor through a plastic bag to avoid absorption of water, making sure that the placed pentoxide was properly dispersed before making subsequent additions, Figure 1a. In reaction PG2, containing less glycerol, the system was heated to 60 °C after the addition of half of the pentoxide because of the increase in the viscosity. In both reactions, to complete the phosphorylation, the system was heated to 80 °C for 8 h. The viscous and transparent beige oils produced were decanted from some burnt particles at the bottom of the reactor.

**Scheme 2:**
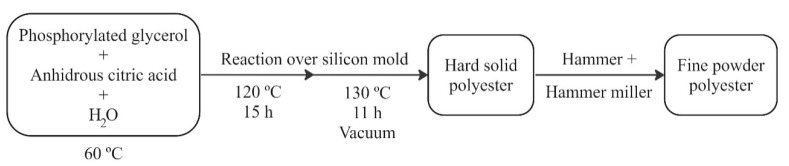
Flowchart of the preparation of phosphate containing glycerol.

Then, to synthesize the cross-linked polyesters (see Scheme 2), 106.3 g of the phosphorylated glycerol samples (PG1 and PG2) were mixed with 112.5 g of anhydrous citric acid and 110 mL of deionized water and stirred at 50 °C to dissolve the acid. Initial tests were performed to determine the ratio of citric acid to phosphorylated glycerol to use. A mass ratio of citric acid to phosphorylated glycerol 1.06 gave the lowest water absorption of the cured polyesters, see complementary data, Figure S1. For polyester without phosphorus (PE0), glycerol was used instead of phosphorylated glycerol, at a 1:1 molar ratio with citric acid. The prepared solutions were casted in open silicone molds (200 × 200 mm). Firstly, they were heated in a ventilated oven at 120 °C for 15 h and thereafter for 11 h in a vacuum oven at 130 °C for further forcing esterification and evaporation of the water from the reaction products. The resulting semitransparent hard solid polyester samples (Figure 1b) were then broken with a hammer before being milled to a fine powder in a hammer mill. Polyester samples PE1 and PE2 were produced with PG1 and PG2 respectively. The powder was kept in airtight containers to avoid moisture absorption.

### 2.3. Particleboard Formation

**Figure 2:**
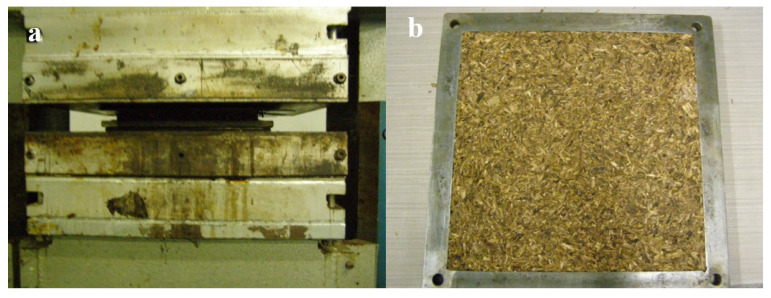
(**a**) Hot pressing of particleboard. (**b**) Pressed board still in the iron mold.

**Scheme 3:**
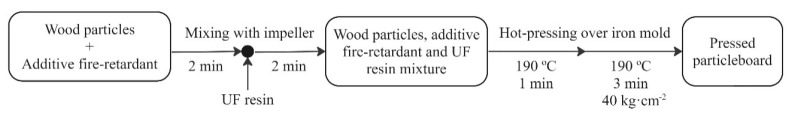
Flowchart of the preparation of FR-containing particleboards.

For the preparation of the laboratory particleboards with the fire retardants (aimed density 650 kg·m^−3^), an iron metal mold (210 × 210 mm^2^, thickness 11 mm) was used and one-layer boards were pressed using dry (12 wt% humidity) softwood particles of 5–30 mm length through compression molding by means of a Cortazar Especial platen press (see Figure 2). The procedure is summarized in Scheme 3. First, the wood particles were mixed with the fire-retardant additives (except the blank sample A, see Table 2) for 2 min with an impeller and then further 2 min after the addition of the UF-resin. The iron-mold was then filled with the help of a spoon and the pressing cycle used was the following: 190 °C with pre-heating of the filled mold for 1 min and then pressing with 40 kg·cm^−2^ for 3 min.

For conditioning the boards before making the experiments, they were placed in a chamber at 23 °C and 50% relative humidity for 1 month.

### 2.4. Measurements

#### 2.4.1. ATR-FTIR Characterization

To characterize the products, ATR-FTIR spectra were recorded on a Nicolet iS5 apparatus from Thermo Scientific, using a diamond iD7 transmission accessory (16 scans, 4 cm^−1^ resolution, 4000–400 cm^−1^). On the other hand, TGA residues were analyzed by Thermo Scientific iS 50 FTIR (32 scans, 8 cm^−1^ resolution and 4000–400 cm^−1^ for middle IR and 32 scans, 4 cm^−1^ resolution and 1800–100 cm^−1^ for far IR).

#### 2.4.2. ^1^H-NMR Characterization

Nuclear magnetic resonance was also employed to characterize the obtained glycol phosphates. For this purpose, Avance 300 DPX (Bruker) NMR (Billerica, MA, USA) was used, and spectra were obtained using deuterated dimethyl sulfoxide (DMSO-d_6_) as solvent.

#### 2.4.3. SEM-EDX

SEM (3030 Hitachi) analysis, followed by energy EDX (Quantax EDS Bruker) (Berlin, Germany) analysis was used to identify the elemental composition of phosphorylated polyesters. The measurements were performed under vacuum and 8 kV voltage.

#### 2.4.4. TGA Analysis

All thermogravimetric tests were performed on a TGA Q500 (TA Instruments, Delaware, United States). For this purpose, 10–15 mg of the sample was heated from 30 °C to 800 °C at a rate of 10 °C·min^−1^ under nitrogen or air atmosphere of 90 mL·min^−1^.

#### 2.4.5. TGA-FTIR Analysis

The TGA evolved compound were analyzed coupling the TGA instrument (TGA Q500 from TA Instruments, EGA furnace) with an Infrared (FTIR) spectrometer (Thermo iS 50 FTIR) by means of a TGA-interface. First, 10–15 mg of the sample was heated from 30 °C to 800 °C at a rate of 10 °C·min^−1^ under nitrogen atmosphere. The temperature of the Thermo Scientific transfer line between the thermobalance and the FTIR gas cell were set at 220 °C. The flow rate of nitrogen in the cell was set at 90 mL·min^−1^ and the spectra in the form of interferograms were continuously recorded in the spectral range of 4000–500 cm^−1^. Afterward, these interferograms were processed to build up the Gram–Schmidt (GS) reconstruction.

#### 2.4.6. Water Swelling Performance and Density Determination

To assess the water swelling of the different samples, a methodology based on the European standard EN 317:1993 was used [26]. After conditioning, the boards were cut into 50 × 50 mm^2^ squares. The initial density was determined after measuring the square dimensions and thickness with a micrometer to calculate the volume of the sample. To calculate the density, the weight of each sample was divided by the volume and the density was given as an average of 4 samples in kg·m^−3^. Thickness swelling, determined as the percentage increase in initial thickness, and water absorption, determined as percentage increase in initial mass, were determined as an average on 4 sample replicates after 24 h of immersion in deionized water at 20 °C.

#### 2.4.7. Cone Calorimeter Tests

The particleboards were cut to obtain 10 × 10 × 1.2 cm^3^ plates. All plates were stored in a conditioning chamber at 23 °C and 50% relative humidity for a month before being tested. Combustion experiments were performed on the plates by means of a cone calorimeter (Fire Testing Technology, FTT), at an incident heat flux of 35 kW·m^−2^, with a retainer to an exposed area of 88.4 cm^2^ and a distance of 25 mm from the heater. The experiments were carried out in a 1200 s time span. Heat Release Rate (HRR, kW·m^−2^), Total Heat Release (THR, MJ·m^−2^), Mass Loss (ML, %), Total Smoke Production (TSP, m^2^) and Specific Extinction Area (SEA, m^2^·kg^−1^) were measured. All tests were performed in triplicate and in accordance with ISO 5660-1:2013.

## 3. Results and Discussion

### 3.1. Phosphorylated Polyester

#### 3.1.1. ATR-FTIR Characterization

**Figure 3:**
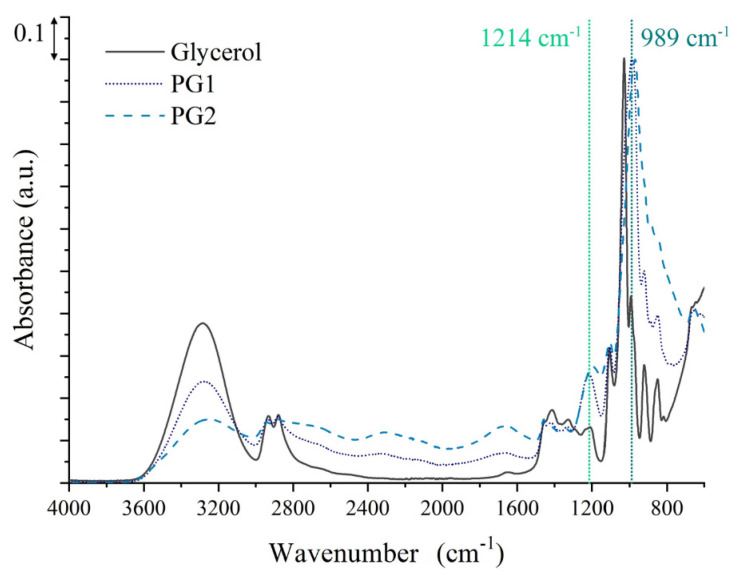
ATR-FTIR spectra of phosphorylated glycerol products PG1 and PG2 and pure glycerol as reference.

**Figure 4:**
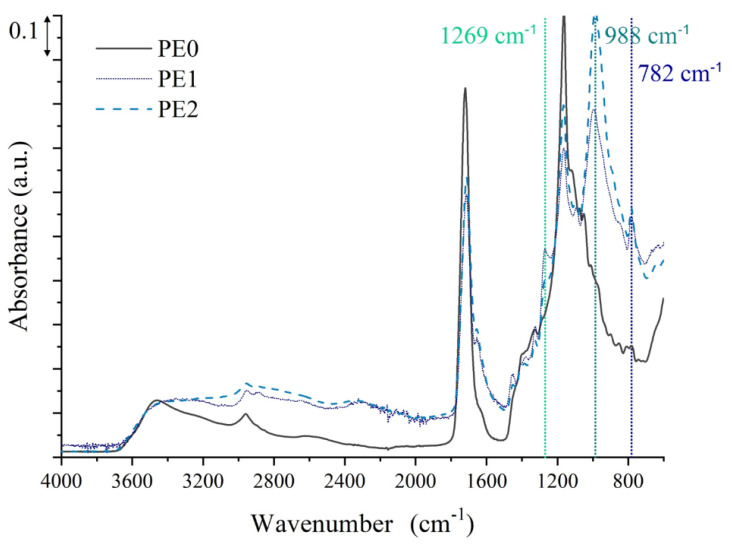
ATR-FTIR spectra of phosphate-containing bio-based polyesters PE1 and PE2 and polyester without phosphate as reference PE0.

The phosphorylated glycerol intermediates and the final solid polyesters were characterized by ATR-FTIR spectroscopy (Figure 3 and Figure 4, respectively). As observed (Figure 3), after phosphorylation of the glycerol, the bands at 1214 cm^−1^ (P=O stretching vibration) and 989 cm^−1^ (P-O stretching vibration) increased, similarly to what was reported by Bocz et al. [25]. These changes confirmed the incorporation of phosphorus to the glycerol structure. In the spectrum of the polymers (Figure 4), the absorptions at 1735 cm^−1^ (C=O stretching) and 1190 cm^−1^ (C-CO-O stretching) corroborated the polyester formation. Moreover, in the samples synthesized from the phosphorylated polyols new bands could be identified at 1269 cm^−1^ (P=O stretching vibration), 988 cm^−1^ (P-O stretching vibration) and 782 cm^−1^ (P-O-C asymmetric stretching vibration) [27]. All these bands verified the occurrence of the polymerization reaction. 

Even though, initially, the polymer was hard and resistant, in contact with water it slowly absorbed water and softened and changed the water pH (<3). Initial tests to neutralize the acidic phosphate groups of the polyester with ammonium or sodium counter ions were discouraging, as it rendered the polyester even more hygroscopic (data shown in the Supplementary Material Table S1).

#### 3.1.2. ^1^H-NMR Characterization

**Figure 5:**
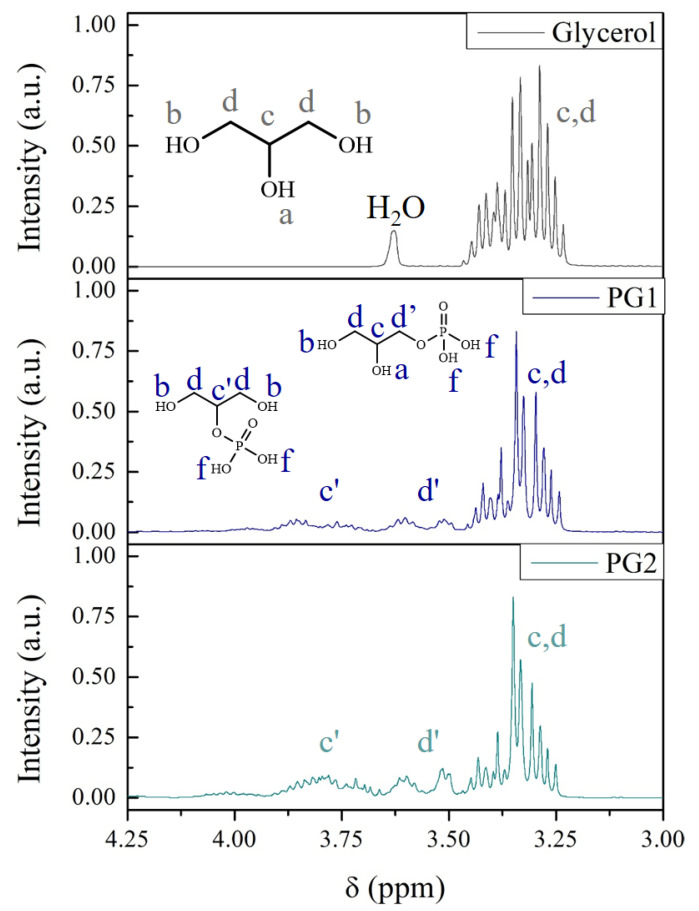
^1^H-NMR spectra of phosphorylated glycerol products PG1, PG2 and pure glycerol as reference.

Likewise, ^1^H-NMR spectra for raw glycerol, PG1 and PG2 are shown in Figure 5. For glycerol, the signals corresponding to the methylene and methine groups appeared at 3.5–3.2 ppm, while the alcohol protons were at 4.6–4.4 ppm (this spectral range is not shown in Figure 5). The spectra of the phosphorylated glycols showed new signals at 4.1–3.7 ppm and 3.7–3.5 ppm, which could be associated with methine and methylene next to phosphorylated alcohol respectively. These results confirmed the successful phosphorylation of glycerol. The area of the new signals was compared to those corresponding to the glycerol methine and methylene groups and it could be observed that by increasing the phosphorus pentoxide/glycerol ratio employed the area of these signals increased, indicating that the phosphorylation degree increased from PG1 to PG2. Further analysis of these results (shown in the Supplementary Materials) led to the observation that an -OH substitution of 34% was obtained for PG1 and 51% for PG2. These results, together with the incorporated glycerol/phosphorus pentoxide ratio of 3:1 for PG1 and 1.5:1 for PG2, led to the conclusion that complete reaction between the glycerol and P_2_O_5_ was obtained.

#### 3.1.3. SEM-EDX

**Figure 6:**
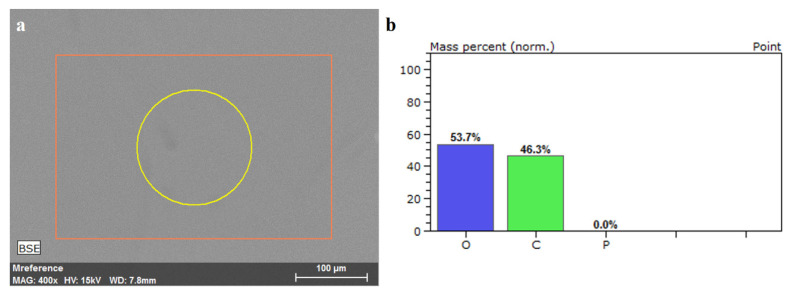
(**a**) SEM analysis and (**b**) EDX analysis of sample PE0.

**Figure 7:**
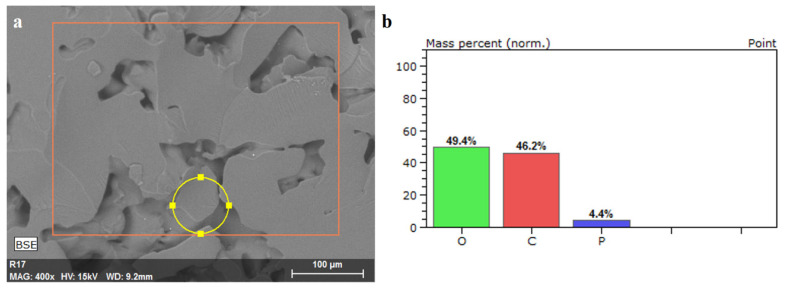
(**a**) SEM analysis and (**b**) EDX analysis of sample PE1.

**Figure 8:**
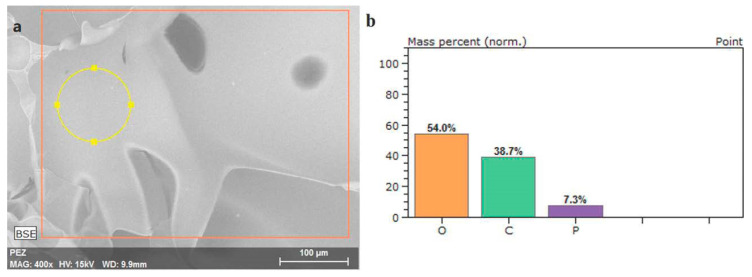
(**a**) SEM analysis and (**b**) EDX analysis of sample PE2.

In order to quantify the phosphorus content of the final polymers, SEM-EDX analyses were carried out, and the results obtained can be found in Figure 6, Figure 7 and Figure 8 for the samples PE0, PE1 and PE2, respectively. As expected, no evidence of phosphorus was found for sample PE0. As for the other samples, the amount of phosphorus found (4.4 wt% and 7.3 wt%) was in line with the results obtained, since for sample PE1, a value of 4.3 wt% of phosphorus was expected, and for sample PE2, 7.2 wt%.

#### 3.1.4. TGA Analysis

**Figure 9:**
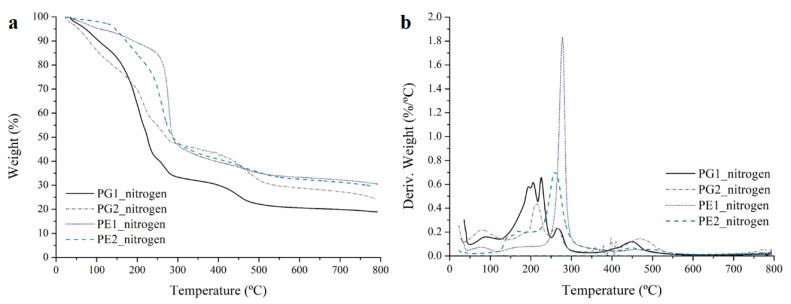
(**a**) TGA and (**b**) DTG data for the different monomers and polymers in nitrogen.

**Figure 10:**
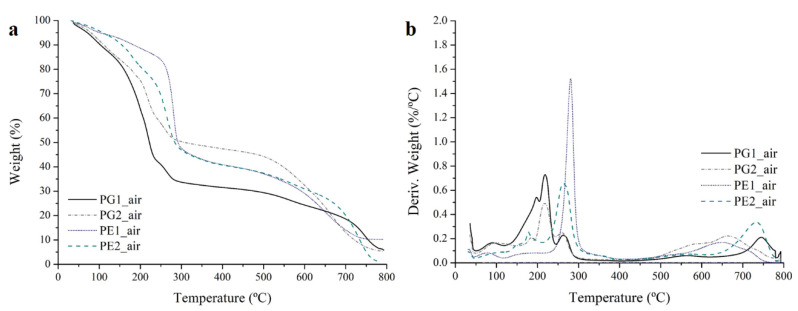
(**a**) TGA and (**b**) DTG data for the different monomers and polymers in air.

TGA measurements were performed under nitrogen and air atmosphere, as shown in Figure 9 and Figure 10, respectively, and the data obtained are summarized in Table 3. As observed, under nitrogen and air atmosphere phosphorylated glycerol samples showed three main weight loss steps, in the range of 50–300 °C, 300–500 °C and 600–800 °C. The first step, which presented a weight loss higher than 50% in all the samples, was related to the phosphorus release [28] and does not leave any residue. The phosphorus release produced the dehydration of the sample giving rise to char formation [28]. The pyrolysis of the uncarbonized segment produced the second weight loss step between 300–500 °C [29]. As expected, the increase in phosphorus content from PG1 to PG2 caused the reduction of the mass loss during the first step together with the increase in the residue under nitrogen [30]. The residue in air was lower than in nitrogen but it remained constant with the phosphorus content.

Notably, the main difference between the two atmospheres was in the final degradation step (600–800 °C). Thus, the weight loss of this step was very low under nitrogen but presented significant values under air. This result was in accordance with literature data where it is stated that the decomposition of the char is favored under thermo-oxidative atmosphere. Accordingly, the final residue in air was lower than the obtained under nitrogen [30,31,32,33]. 

Phosphorylated polymer samples showed the same weight loss pattern, with three main weight loss steps. However, the onset of the first step was delayed in the polyesters, because of their crosslinked nature. In addition, the residue under nitrogen of the polymer samples was higher than the obtained for the phosphorylated polyols. In the literature, it is described that the polyester formed from glycerol and citric acid presents two degradation stages [33], the last one being attributed to the degradation of aromatic moieties from the cyclization of citric acid [34]. This effect could be in the origin of the higher char percentage produced in the polyesters. Under oxidizing atmosphere, the main difference was again in the last weight loss step related to the char decomposition that was significant under air and yielded low residue values. 

#### 3.1.5. TGA-FTIR Analysis

**Figure 11:**
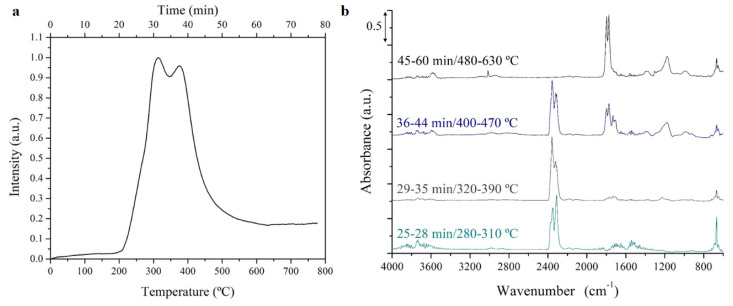
(**a**) Gram–Schmidt over time and temperature and (**b**) FTIR spectra recorded at different intervals for PE0 sample.

The gases produced in the thermogravimetry under nitrogen were analyzed by infrared spectroscopy using the TGA-FTIR coupling. The Gram–Schmidt chromatogram of the non-phosphorylated polymer, that can be related to the derivative TG curve, is shown in Figure 11a. According to the data, four degradation steps were appreciable. Infrared spectra registered in the different steps are shown in Figure 11b. The spectrum obtained in the first step between 25–28 min (280–310 °C) presented bands at 4000–3400 cm^−1^ (O-H stretching vibration) and 1800–1300 cm^−1^ (H-O-H bending vibration), related to water release [35,36,37]. CO_2_ was also detected by the bands at 2400–2250 cm^−1^ (C=O asymmetrical stretching vibration) and 750–600 cm^−1^ (O=C=O bending vibration) [36,38,39], and bands due to CO (2181–2118 cm^−1^), which were related to the ester group decarboxylation. In the second step, between 29 and 35 min (320–390 °C), in addition to the bands due to carbon dioxide, new absorptions appeared at 2886–2626 cm^−1^ and 1749–1687 cm^−1^ that were related to the presence of acrolein. Acrolein could be originated from glycerol dehydrogenation as stated in literature by R. Estebez et al. [39]. In the third step, another absorption was observed in the carbonyl stretching (1830–1750 cm^−1^) that was related to the evolved acetic acid. Finally, in the last step, 45–60 min (480–630 °C), methane was released, as shown by the bands at 3015 cm^−1^ (C-H stretching vibration of alkane groups) [40,41,42], which could correspond with the char degradation.

**Figure 12:**
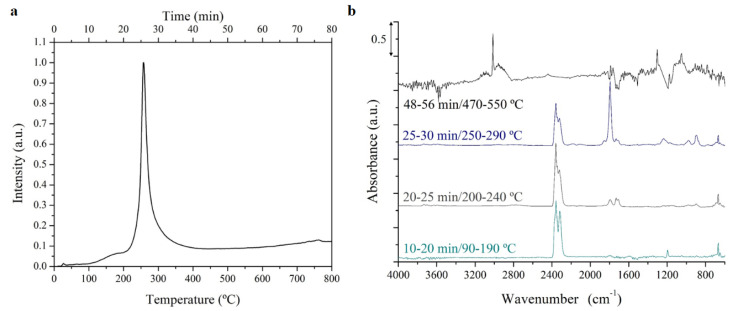
(**a**) Gram–Schmidt over time and temperature and (**b**) FTIR spectra recorded at different intervals for PE1 sample.

**Figure 13:**
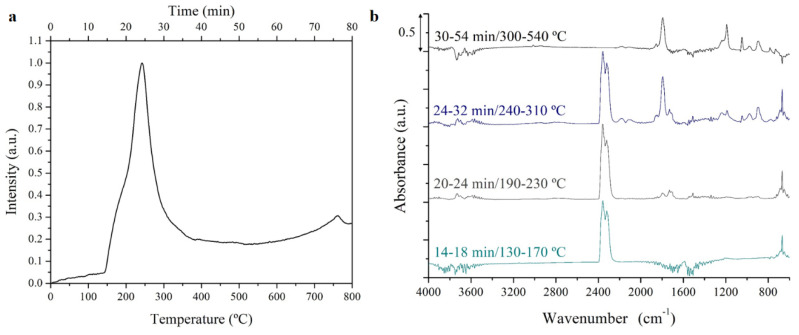
(**a**) Gram–Schmidt over time and temperature and (**b**) FTIR spectra recorded at different intervals for PE2 sample.

Likewise, the results obtained for the phosphate containing polyesters PE1 and PE2 are shown in Figure 12 and Figure 13, respectively. According to the data obtained, in the early stages of degradation water and CO_2_ signals were appreciable. Afterward, as for reference polyester, signals from acrolein originated from glycerol dehydrogenation were detected [39]. In the next step, citraconic anhydride was observable by the band at 1840 cm^−1^ and 1765 cm^−1^ (C=O stretching of anhydride compounds) and 920 cm^−1^ (C-O stretching of the C-O-C) [43,44]. According to the literature, this anhydride is formed from the decomposition of citric acid [43]. Finally, at high temperature, methane was released.

It is worth mentioning that with the degradation of both phosphorylated polyesters, citraconic anhydride was detected instead of acetic acid, which was one of the gases evoling with degradation of the non-phosphorylated sample. This effect could be related to the dehydration of the sample produced by the phosphorus release [44].

**Figure 14:**
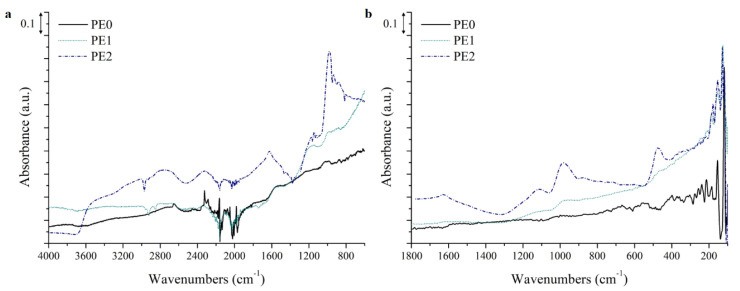
(**a**) Middle and (**b**) far ATR-FTIR spectra of the TGA-FTIR residues.

As no phosphorus signal was appreciable in the compounds released during degradation, the final residues in nitrogen were analyzed by middle and far ATR-FTIR and the spectra are shown in Figure 14. For sample PE0, only weak signals were appreciable at about 3015 cm^−1^ (CH_2_ C-H stretching vibration), 2800 cm^−1^ (CH_3_ C-H stretching vibration), 1550 cm^−1^ (aromatic ring C=C stretching vibration), 1250 cm^−1^ (C-C stretching vibration) and 950 cm^−1^ (aromatic ring C-H out-of-plane deformation) [45]. This could suggest that the sample was almost completely decomposed and that very small amounts of carbonaceous char were obtained [46]. When incorporating phosphorus to the polymer, for sample PE1 small differences were appreciable in mid-FTIR. The main difference regarding PE0 was in the signals at 1200 cm^−1^ and 1020 cm^−1^, which corresponded to P=O and P-O stretching vibrations respectively. As a consequence of its higher phosphorus content, these signals were stronger in the spectra of PE2. Additionally, in this sample, in the far region a signal at 475 cm^−1^ could be appreciated. This signal corresponded to the C-O bending vibration [47]. All these absorptions corroborated the presence of phosphorus in the residue and confirmed that the phosphorus additives acted in the condensed phase.

### 3.2. Particle Board Performances

#### 3.2.1. Water Swelling and Absorption

**Figure 15:**
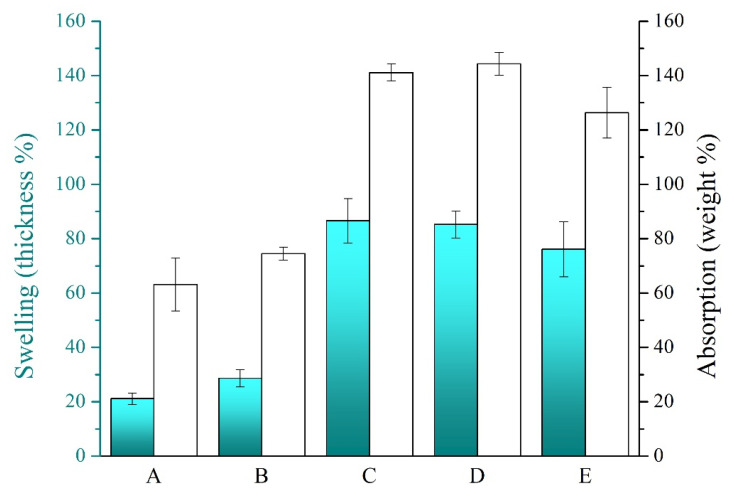
Percentages of thickness swelling (blue) and water absorption (white) of particleboard samples with and without additives.

Samples PE1 and PE2 and commercial ammonium polyphosphate were used to prepare lab particleboards that were immersed in deionized water for 24 h and the swelling and water absorption values were measured (Figure 15). Unfortunately, samples containing both phosphorylated polyester additives showed much higher water swelling and absorption compared to the blank sample A and the APP added sample B. This could be explained on the one hand by the high hygroscopic character of the added polyesters but on the other hand by the acidity of the phosphate groups which could catalyze the UF-resin polymerization even at room temperature. Actually, orthophosphoric acid has been studied as a catalyst for UF-resins and a decreased gelling time occurred with the amount of phosphoric acid added [48]. During the mixing of the components, it was noted that some gelling occurred, which reduced the adhesives wetting of all the wood particles and the resulting boards become badly glued, even causing the dislodging of some fibers during immersion in water. It should be mentioned that no paraffin was added, as in commercial boards, to reduce water absorption and swelling.

#### 3.2.2. Cone Calorimeter Test

**Figure 16:**
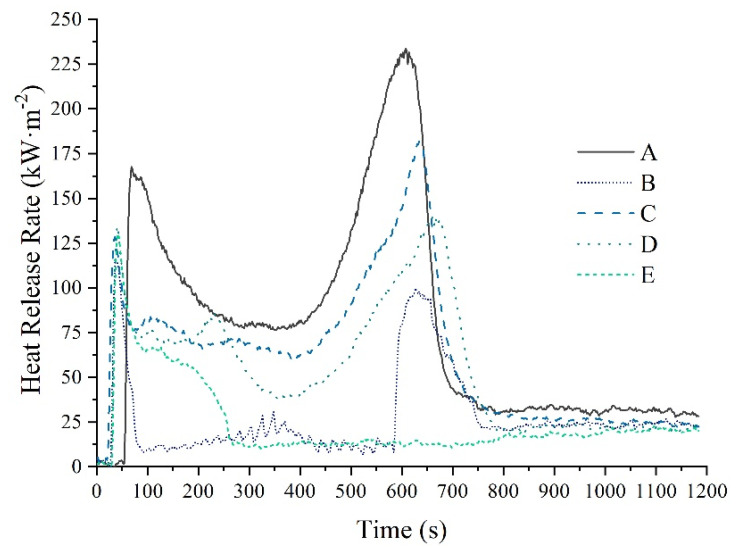
Heat Release Rate (HRR) *versus* time curves for all the samples.

The fire behavior of lab particleboards containing PE1, PE2 and commercial ammonium polyphosphate were analyzed using a cone calorimeter. Figure 16 shows the Heat Release Rate (HRR) vs. time curves for all the samples and the main parameters are summarized in Table 4. Wood materials usually possess a double peak feature. At the beginning of the burning, a sharp peak is observed, which decreases as burning proceeds as a char layer forms on the surface of the wood. Once the char layer is formed gradual burning of the sample though the thickness occurred, followed by the second peak corresponding to the increased rate of the volatiles in the thin unburned part of the sample *prior* to the end of flame burning [49,50]. Flame burning is the most important parameter during the growth and full-developed phase, and it corresponds to the intensity of the fire; meanwhile, fire resistance issues are related to the glowing and are given in the last phase of the combustion. Flame retardants are responsible for the decrease in the midrange intensity (gradual burning across thickness) and the decrease in the second peak [51]. The burning behavior differs from wood specie to specie, e.g., A. I. Bartlett et al. tested several wood species and observed that redwood char formation was 20% faster than southern pine, while basswood samples charred 60% faster than red oak samples [50]. However, engineered wood is supposed to differ from natural wood due to the presence of the resin, absence of grain direction, density and porosity gradient. The employed resin is considered to affect the kinetic mode and heat reactions, due to the fact that the chemical composition and largely their microstructure affects the final particleboard fire characteristics [4]. It should be mentioned that UF-resin has a high amount of nitrogen [52], which is considered to be a flame-retardant element [53]. Porosity which is affected by the particleboard density gives rise to low thermal conductivity, which in turn results in higher thermal capacity and specific heat [54]. As particleboards are engineered, the obtained surface finish is also important to take into account, as P.N. Alexiou et al. mentioned in their work textured surfaces yield lower spread of flame and heat evolution indices than smooth surfaces [55]. Moisture content is also a factor that affects the fire behavior of wood, as after heating and before pyrolysis free water begins to evaporate and the energy is employed for water evaporation rather than heating, so it cools the pyrolysis zone and it takes longer for the sample to burn out [50].

Regarding the results for the obtained particleboards in this work, HRR *vs.* time curves showed the characteristic wood double peak. In more detail, the time of ignition decreased in presence of phosphorus. However, for the phosphorus containing samples, a decrease in the t_ig_ with the phosphorus content was observed. The reduction of the time to ignition produced by the phosphorus was attributed to the low P-O and P-C linkages strength (P-O bond requires of 149 kJ‧mol^−1^ while C-O bond 256 kJ‧mol^−1^ [56,57,58] and P-C bonds are reported to cleave more easily than C-C bonds [58]). 

It is worth mentioning that phosphorus introduction dramatically changed heat emission data of the boards. Specifically, the THR, PHRR and MAHRE were considerably reduced with the phosphorus content. On the other hand, the time at which the PHRR was reached (t_peak_) was lower in samples with high phosphorus content (samples B and E). This result was a consequence of the reduction or disappearance of the second HRR peak (Figure 16). It is well known that after a first ignition, high phosphorus amount causes the flame to disappear and the carbonization of the sample takes place slowly and progressively, generating residue (char) [32].

In accordance with the final residue, as expected, the percentage increases with the phosphorus content [30]. 

**Figure 17:**
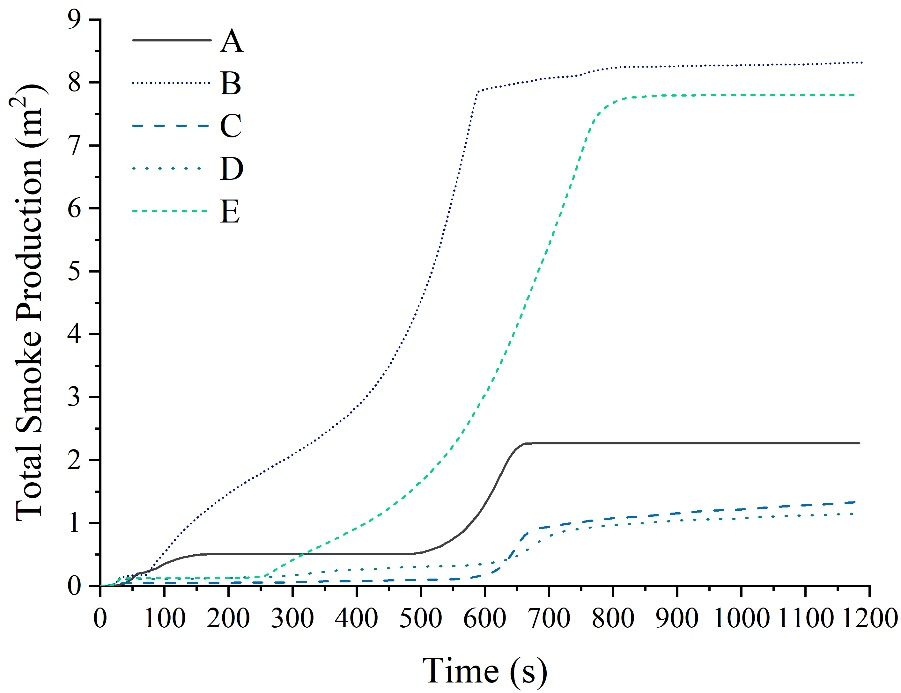
Total Smoke Production *versus* time for all the samples.

Finally, samples containing more phosphorus (B and C) showed higher smoke production (Figure 17) and therefore TSP and SEA values were the highest in these two samples. This behavior was related to the action of the phosphorus by inhibiting the combustion in the gas-phase [59].

According to the cone calorimeter results, phosphorus retardant treatment was effective to protect the boards. The additives acted in the condensed phase, generating a greater amount of char and in the gas phase, inhibiting the formation of flame. The obtained results showed that the fire performance was dependent on the phosphorus content and the behavior of the synthesized polyesters PE1 and PE2 was similar to that of commercial ammonium polyphosphate.

## 4. Conclusions

A new phosphate-containing bio-polyester based on glycerol and citric acid was synthesized and evaluated as a fire retardant in wooden particleboards. The incorporation of phosphate ester in the glycerol monomer was confirmed by ATR-FTIR and ^1^H-NMR. After esterification of the phosphorylated glycerol with citric acid, the thermal degradation of the cured polyesters was determined by TGA in nitrogen and air atmosphere. It was found that the degradation proceeded in three steps, and an increased amount of residual char was produced depending on the phosphorus amount. Wooden particleboards with added bio-polyester FR showed a bad swelling and water absorption behavior, making further optimizations necessary, even though positive results were seen in cone calorimeter tests. More specifically, the THR, PHRR and MAHRE were considerably reduced in presence of the highest phosphate-containing bio-polyester, as well as with the APP reference FR.

## Figures and Tables

**Table 1:** Some examples of incorporation of phosphorus to bio-based resins.

Polymer	Flame Retardant Introduction Mechanism	Bio-Compound	Source
PLA	Phytic acid incorporation by pad-dry-cure technique	Phytic acid and PLA	[15]
PLA	Lignin phosphorylation and conversion to ammonium phosphate	Lignin and PLA	[16]
PHFC	CEPPA esterification with HDO and FDCA	FDCA	[17]
PBT	Epoxidized linseed oil and downstream corn oil phosphorylation	Linseed oil and downstream corn oil	[18]
PU-foam	Epoxidized soybean oil phosphorylation	Soybean oil	[19]
PU-foam	Polyol based on phenylphosphonic acid and propylene oxide	Limonene	[20]
Epoxy resin	Vanillin coupling with diethyl phosphite	Vanillin	[21]
Epoxy resin	Vanillin and guaiacol coupling with DOPO	Vanillin and guaiacol	[22]
Epoxy resin	Epoxy monomer modification with phloroglucinol	Phloroglucinol	[23]
PS	Isosorbide reaction with diphenylchlorophosphate and vinyl group incorporation with acryloyl chloride	Isosorbide	[24]

**Table 2:** Composition of the different mixtures used for lab particleboards and final thickness and density.

Board	Wood Particles [g]	UF-Resin [g]	Additive/Weight [g]	Thickness [mm]	Average Density [kg·m^−3^]
A	304.4	72.74	-	10.9 ± 0.07	658 ± 50
B	250.7	72.74	APP/47.28	10.8 ± 0.03	643 ± 31
C	250.7	72.74	PE1/47.28	11.7 ± 0.12	532 ± 63
D	250.7	72.74	PE2/47.28	11.5 ± 0.14	566 ± 35
E	197.0	72.74	PE2/94.56	11.0 ± 0.13	617 ± 80
F *	197.0	72.74	PE2/94.56	10.4 ± 0.11	626 ± 28

* Same sample as E, but the polyester additive was neutralized with NH_4_OH.

**Table 3:** Data obtained from TGA results.

Sample	Weight Loss [wt%]						Residue at 800 °C [%]	
	50–300 °C		300–600 °C		600–800 °C			
	N_2_	Air	N_2_	Air	N_2_	Air	N_2_	Air
PG1	64.1	63.7	12.8	9.4	1.8	18.5	18.8	6.2
PG2	48.7	47.8	18.3	18.0	4.6	27.2	24.7	5.4
PE1	51.9	51.0	13.4	18.6	2.8	18.8	30.7	10.3
PE2	51.5	52.0	15.2	15.9	3.2	29.9	29.5	1.2

**Table 4:** Cone calorimetry data obtained for all samples.

Sample	P [%]	t_ig_ [s]	PHRR [kW·m^−2^]	t_peak_ [s]	THR [MJ·m^−2^]	MAHRE [kW·m^−2^]	Residue [%]	TSP [m^2^]	SEA [m^2^·kg^−1^]
A	0	49.0	228.2	579.3	92.6	113.7	16.6	1.9	11.8
B	3.95	31.3	114.8	42.0	23.1	43.3	32.9	10.8	233.3
C	0.55	22.5	165.0	632.0	70.7	83.8	20.2	1.1	16.3
D	0.92	28.0	142.7	653.0	65.2	74.6	23.3	0.9	12.3
E	1.87	30.0	134.2	41.0	31.1	61.2	27.5	7.5	141.7

P: theoretically calculated phosphorus amount for each particleboard. t_ig_: time to ignition. PHRR: Peak of Heat Release Rate. t_peak_: time at which the PHRR is given. THR: Total Heat Release. MAHRE: Maximum Average of the Rate of Heat Emission. TSP: Total Smoke Production. SEA: Specific Extinction Area.

## Supplementary Materials

The following supporting information can be downloaded at: https://www.mdpi.com/article/10.3390/polym15051093/s1, Scheme S1: Phosphate containing polyester synthesis reaction; Figure S1: Water absorption of cured polyesters with phosphorylated glycerol: PG1 and PG2; Table S1: Swelling and water absorption data for particleboard F; measurements of neutralization of acidic phosphate groups; glycerol conversion measurements from ^1^H-NMR; Table S2. Data obtained from ^1^H-NMR.
