# Supplementary material for: Bio-Based Phosphate-Containing Polyester for Improvement of Fire Reaction in Wooden Particleboard
Source: Polymers (Basel). 2023 Feb 22;15(5):1093. doi: 10.3390/polym15051093 (PMC10007242; doi:10.3390/polym15051093)
Supplement: Supplementary file 1 [file polymers-15-01093-s001.zip › polymers-2213169-supplementary.pdf]

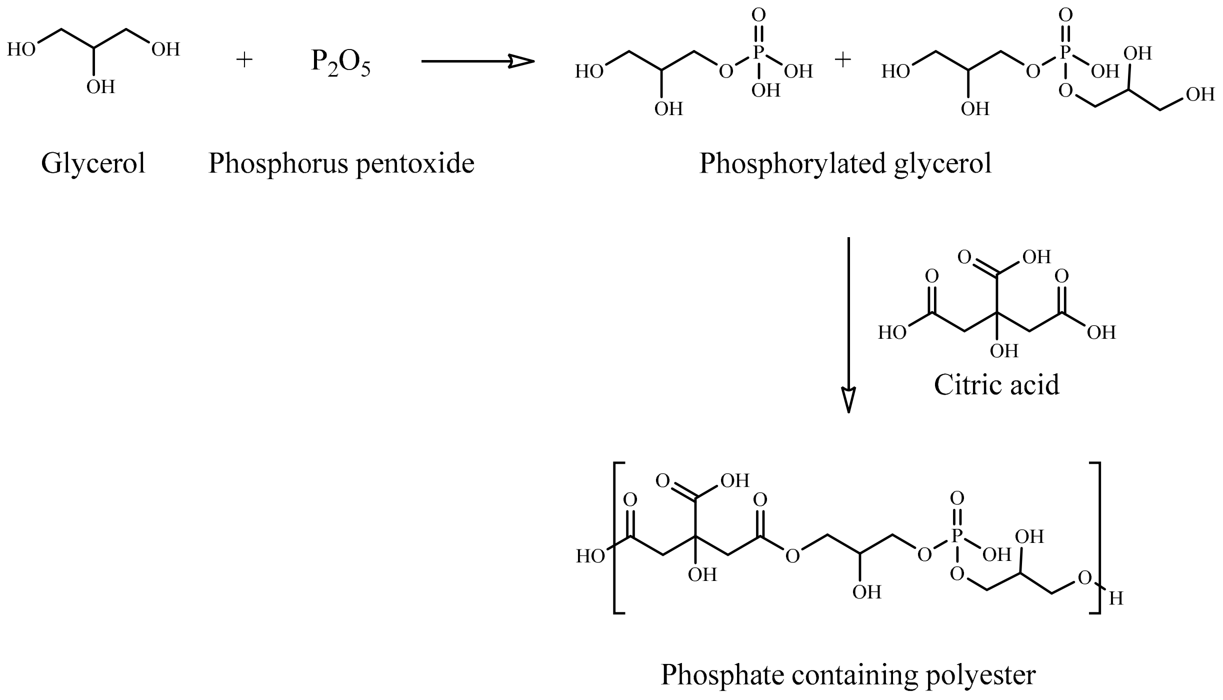

Scheme S1. Phosphate containing polyester synthesis reaction.

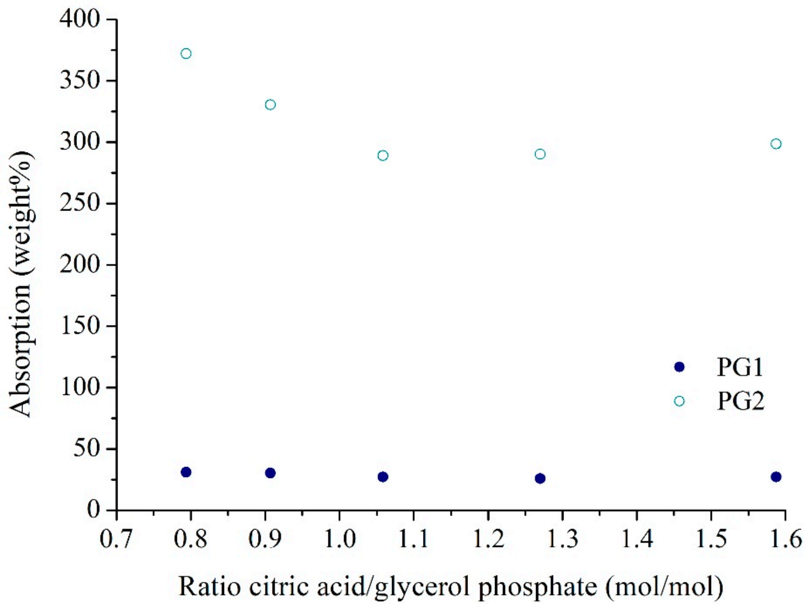

Figure S1. Water absorption of cured polyesters with phosphorylated glycerol: PG1 and PG2.

Table S1. Swelling and water absorption data for particleboard F

Sample	Swelling [Thickness%]	Absorption [Weight%]
**F**	74 ± 7	167 ± 14

From the ^{1}H-NMR data collected in **Table S2**, it was possible to calculate de mol% of phosphorylated and non-phosphorylated carbon atoms as follows:

If, P_s (parts per unit of substituted secondary alcohols) can be calculated according to **Equation (S1)**, non-substituted secondary alcohols would be 1-P_s, correspondingly P_s would match the non-substituted primary alcohols. Hence, it was possible to calculate the overall non-substituted alcohols (P_{c+d}) by means of **Equation (S2)**. And from this, the percentage of substituted carbons can be calculated by **Equation (S3)**.

$$P_s = \frac{A_{c'}}{{A_{d'}}/{2} + A_{c'}} \quad \textbf{Equation (S1)}$$

$$P_{c+d} = 2 * P_p + (1 - P_p) \quad \textbf{Equation (S2)}$$

$$\text{Percentage of substituted carbons} = \frac{A_{c'} + {A_{d'}}/{2}}{A_{c'} + {A_{d'}}/{2} + {A_{c+d}}/{P_{c+d}}} * 100 \quad \textbf{Equation (S3)}$$

Table S2. Data obtained from ^{1}H-NMR.

Sample	Area$_{c'}$ [3.88–3.66 ppm]	Area$_{d'}$ [3.66–3.48 ppm]	Area$_{c+d}$ [3.48–3.20 ppm]	-OH Substitution [%]
**PG1**	0.59	0.41	2.66	34
**PG2**	0.62	0.37	1.37	51
